# Editorial: Community Series in Engineered Immune Cells in Cancer Immunotherapy (EICCI), volume II

**DOI:** 10.3389/fimmu.2026.1833581

**Published:** 2026-04-20

**Authors:** Axel Schambach, Francisco Martin, Cristina Maccalli

**Affiliations:** 1 Institute of Experimental Hematology, Hannover Medical School, Hannover, Germany; 2 Division of Hematology/Oncology, Boston Children’s Hospital, Department of Pediatric Oncology, Dana-Farber Cancer Institute, Harvard Medical School, Boston, MA, United States; 3 GENYO, Centre for Genomics and Oncological Research, Pfizer, University of Granada, Andalusian Regional Government, Parque Tecnológico Salud (PTS) Granada, Granada, Spain; 4 Instituto de Investigación Biosanitaria ibs.GRANADA, Granada, Spain; 5 Department of Biochemistry and Molecular Biology III and Immunology, Faculty of Medicine, Excellence Research Unit “Modeling Nature” (MNat), University of Granada, Granada, Spain; 6 IRCCS Azienda Ospedaliera Metropolitana Plesso Policlinico San Martino, Genoa, Italy

**Keywords:** adoptive cell therapy (ACT), allogeneic cell therapies, chimeric antigen receptor, gene editing, tumor-associated antigens

In recent decades, our understanding of adoptive cell therapy (ACT) and the genetic modifications of lymphocytes and other immune cells to enable the external expression of receptors targeting tumor antigens has significantly advanced and accelerated the clinical development of this strategy. CD19-targeting T cells engineered with the Chimeric Antigen Receptor (CAR) have achieved 50–90% complete response rates in refractory B-cell malignancies, leading to regulatory approval for seven “living drugs.” Despite this success, autologous manufacturing remains complex and lengthy, limiting the number of patients who can benefit from this approach. Toxicities associated with the treatment, such as cytokine release syndrome (CRS) and immune effector cell-associated neurotoxicity syndrome (ICANS), remain among the major challenges, in addition to the limited clinical efficacy for the treatment of solid tumors.

To address these limitations, current research focuses on developing novel strategies designed to improve treatment accessibility, mitigate adverse effects, and enhance anti-tumor activity in solid malignancies. The “*Community Series in Engineered Immune Cells in Cancer Immunotherapy (EICCI), volume II*” offers a comprehensive overview of the most recent advances and persistent challenges in the field of adoptive cell therapy. Throughout this Research Topic, cutting-edge strategies designed to overcome the limitations of current treatments are explored, ranging from the development of universal ‘off-the-shelf’ allogeneic therapies using gene-editing tools, to the engineering of alternative cellular platforms such as NK cells, macrophages, and neutrophils (CAR-NK, CAR-M, CAR-N). Furthermore, the series delves into efforts to conquer the hostile solid tumor microenvironment, the identification of novel target antigens for resistant hematological malignancies, the optimization of clinical administration routes, and the molecular understanding for a safer and more effective management of associated toxicities, altogether drawing the roadmap toward the next generation of ‘living drugs’.”

## Allogeneic/universal (U-)CAR-T cells

### The shift from autologous to allogeneic platforms

The approved living drugs focus on a personalized approach utilizing patients’ T lymphocytes to produce CAR-T cell therapies. This method faces several challenges, including low yields of circulating T cells (particularly in heavily pre-treated or pediatric patients) inefficient *ex vivo* proliferation of the immune cells, and the relatively lengthy manufacturing process that might result in treatment failure, especially for patients with rapid disease progression. To address these limitations, allogeneic CAR-engineered immune cells are created using donor peripheral blood mononuclear cells (PBMCs) (1, 2). This approach reduces dependence on patients’ cell availability, lowers production costs, and increases accessibility for larger numbers of cancer patients. Several groups worldwide are investigating allogeneic/universal-engineered immune cells, utilizing different sources of lymphocytes (e.g., PBMC, umbilical cord blood, induced pluripotent stem cells (iPSC), etc.) with ongoing clinical trials assessing their safety and effectiveness (1, 2). However, conclusive data regarding the optimal source of lymphocytes are not available. Moreover, these strategies have to consider the risk of graft-versus-host disease (GvHD), due to HLA mismatches, upon the infusion of the living drugs into patients.

### Gene-editing strategies for universal CAR-T cells

To prevent the risk of GvHD, genome editing techniques such as CRISPR/Cas9 and TALEN are employed to knock out molecules mediating allogeneic reactivities (e.g., T cell receptors (TCR), CD52, and β2-microglobulin) (3–6). These methods create double-strand breaks in the genome, which are repaired through non-homologous end-joining or homologous recombination, allowing for targeted modifications (3–6). These approaches can overcome the risk of allogeneic reactions *in vivo* but can be associated with the risk of off-target INDELs and imply extensive genetic manipulation of the cellular products. In this direction, Pavlovic et al., describe the generation of universal CD19 CAR T cells through the CRISPR/Cas9 editing of HLA-I and TCR (7). The on- and off-target assessment in CRISPR-Cas9 edited cells was determined by RNA sequencing. The study demonstrates that the selection of the memory phenotype of T cells with a 1:1 ratio of CD8^+^ and CD4^+^ T cells implements long-term anti-tumor activity and is associated with a low pro-inflammatory profile. The drawback of the study was the limited *in vitro* expansion of the pre-selected CAR-T cells to allow the validation of the results in *in vivo* models and their translational application. The obtained achievements support future investigations into the optimization of the production of U-CAR-T to enhance their anti-tumor activity and safety (7). Beyond optimizing universal CAR-T cells, Maldonado-Perez et al., developed TCR-knockout (TCRKO) CD19-ARI-0001cells (CD19-ARI-0001 received Spanish Hospital Exemption for treating adult relapsed/refractory B- Acute Lymphoblastic Leukemia (ALL). By utilizing TRAC gene deletions, the researchers successfully eliminate allogeneic reactivity without compromising the cells’ anti-tumor potency or mitochondrial metabolism. The resulting product maintaines a stable T-cell physiology and a favorable phenotype, composed primarily of effector memory (EM) and 10–20% stem cell memory (TSCM) T cells (8). This demonstrates that large-scale gene editing can produce a safe, effective, and persistent allogeneic therapy for B-cell malignancies that is physiologically comparable to wild-type constructs.

### Pioneering myeloid and alternative lymphoid platforms

The emerging role of macrophages and neutrophils in ACT, specifically CAR-engineered macrophages (CAR-Ms) and neutrophils (CAR-Ns), is documented by Liang et al., who highlight design strategies alongside preclinical and clinical evidence (9). These products offer distinct advantages for solid tumor treatment, such as improved infiltration, modulation of the immunosuppressive tumor microenvironment, and activation of antitumor immunity. However, they remain in early development, facing hurdles in safety, scalability, persistence, and cytokine-mediated toxicities. While iPSCs or hematopoietic stem cells (HSPCs) are appealing sources for these cells, M2-like peritoneal macrophages from gastric cancer ascites can also be engineered with CAR constructs to adopt an M1-like, pro-inflammatory phenotype that enhances T-cell proliferation (9). Addressing these issues remains critical to realize the therapeutic potential of these myeloid platforms.

Finally, dissecting the biological features of αβ- T and γδ-T, NK, and iNKT cells is essential for identifying the optimal cellular subtype for specific therapies. Kent et al., provide an overview of these subtypes in the context of Acute Myeloid Leukemia (AML), focusing on their antigen-dependent and independent activation mechanisms (10). While the majority of ACT studies in AML currently utilize αβ-T lymphocytes, increasing functional and molecular knowledge of NK, iNKT, and γδ-T cells is successfully redirecting scientific attention toward these alternative immune cell platforms (10).

### The rising potential of CAR-NK cells

Although NK, iNKT, and γδ-T cells are less abundant in the circulating blood, the technology for engineering and expansion has been improved and optimized for cell therapy applications. They represent truly “off-the-shelf” cellular products, due to the lack of HLA-restriction and the inability to induce GvHD. The major limitation of these approaches is the limited *ex vivo* expansion of these types of cells and their survival and persistence *in vivo*. Further efforts are required to understand the mechanisms of expansion and activation and which subpopulation of cells could be selected as the optimal allogeneic “living drug”. Along this line, CAR-NK studies appeared in 1028 publications in 346 journals from 5371 authors from 65 Countries with a significant increase in numbers from 2018 to 2021, as reported by Zhang et al. (11). The bibliometric study has been performed using CiteSpace and VOSviewer and highlights the increasing interest in developing CAR-NK-based cell therapies for both hematological and solid tumors by different research teams, with the University of Texas MD Anderson Cancer Center in the US representing the most proficient in the field. The report is an interesting summary of the advances in CAR-NK technological platforms and the related clinical studies. This study concludes that several aspects still need to be implemented and require further research efforts: 1. efficiency and *ex vivo* expansion and persistence *in vivo*; 2. optimization of CAR structure to increase the anti-tumor properties; and 3. the application of gene editing to potentiate the features of CAR-NK cells (11).

## The application of CAR-T cells in solid tumors

### Barriers in the solid tumor microenvironment: enhancing precision, trafficking, and safety

While CAR-T therapies have revolutionized hematological malignancy treatment, their efficacy in solid tumors remains limited by the immunosuppressive tumor microenvironment (TME), poor cell trafficking, and low or heterogeneous antigen expression. Nasiri et al., reviewed these challenges in the context of ovarian cancer (OC) (12) and triple-negative breast (TNB) cancer (13), noting that despite targeting over 30 antigens, preclinical success hasn’t yet translated into striking clinical outcomes. To improve the therapies for these types of tumors, researchers are prioritizing the identification of optimal tumor-associated antigens (TAAs) while refining safety through moderate-affinity epitopes, suicide switches, and transient mRNA-based expression (12). This includes identifying and combining multiple target antigens (such as mesothelin, which is relevant in both pathologies), designing “armed” CARs capable of secreting cytokines to counteract immunosuppression, and optimizing cell trafficking. The goal is to transform this promising cellular therapy into a truly effective clinical tool against these tumors.

Precision targeting of cell therapies is further enhanced by combining CAR-T cells with chemotherapy to upregulate TAAs or co-engineering them with chemokine receptors to improve site-specific migration. The design of CD40L-modified CARs also show promise by directly attacking tumor cells while simultaneously activating antigen-presenting cells (APCs) to engage broader immune subpopulations (12). Ultimately, achieving success in these solid malignances requires a dual focus on overcoming immune evasion and refining antigen specificity, positioning CAR-T therapy as a potent adjunct to conventional treatments once these technical hurdles are resolved (13). Until these technical challenges are fully resolved, CAR-T therapy is expected to serve as a powerful adjunct to be integrated with conventional treatment modalities.

## Novel target antigens for engineered immune cell therapies

Maximizing the efficacy and precision of cell therapies relies on identifying novel target molecules expressed almost exclusively by cancer cells. This high specificity is essential to prevent off-target effects and severe toxicities. Such unintended interactions are particularly dangerous when they affect vital organs essential for patient survival, or when they target the therapeutic T cells themselves, leading to fratricide.

### Overcoming hurdles in T-cell malignancies: multi-targeting and advanced engineering

Despite these advances, autologous CAR-T therapy for T-cell malignancies faces hurdles like T-cell aplasia, product contamination, fratricide killing and tumor heterogeneity. To address these, advanced engineering, such as multi-antigen targeting and “off/on” switches, is essential. The review by To et al. (14) explores the development and challenges of chimeric antigen receptor (CAR)-T cell therapy for treating cutaneous T cell lymphoma (CTCL), a type of non-Hodgkin lymphoma. To overcome these hurdles, the authors highlight advanced engineering strategies, such as multi-antigen targeting utilizing dual CARs and logic gating systems (e.g., “HELP”, “OR”, and “NOT” gates) targeting specific antigens like CD4, CD47, and CD30. Other proposed solutions include utilizing transient CARs, incorporating safety “on/off” switches to manage toxicity, and purifying CD8+ T cells to avoid product contamination with malignant CD4+ T cells. Ultimately, these combinatorial and optimized target strategies aim to strike a vital balance between maximizing anti-tumor efficacy and preserving patient safety in CTCL therapies.

In the same direction, Ibañez-Navarro et al. (15), present an original article showing that the development of NKG2D-CAR memory T cells represents a promising milestone in the immunotherapy landscape for pediatric refractory and relapsed T-cell acute lymphoblastic leukemia (T-ALL). These engineered cells demonstrate robust cytotoxicity against leukemic blasts *in vitro* and successfully delay tumor progression while prolonging survival *in vivo*. Notably, they retain anti-leukemic activity even in the presence of immunosuppressive soluble NKG2D ligands that are shed by the tumor (16, 17). However, the ultimate curative potential of this therapy as a standalone treatment is hindered by its failure to eradicate the leukemia-initiating cell (LIC) compartment. Because the treatment paradoxically triggers the upregulation of genes associated with cancer stem cells in the surviving leukemic cells, disease relapse eventually occurs. Consequently, while NKG2D-CAR T cells provide a highly potent mechanism for bulk tumor reduction, realizing their full clinical promise will require combinatorial strategies. Future therapeutic protocols must pair these advanced CAR-T cells with targeted agents specifically designed to dismantle the immune-resistant LIC subpopulation, thereby preventing relapse and paving the way for a definitive cure.

### Expanding the antigen repertoire: CD176 and CD38 as therapeutic targets

To overcome the limitations of current immunotherapies, Dragon et al. (18), explore the pan-tumor carbohydrate antigen CD176 (Thomsen-Friedenreich antigen), a pan-tumor antigen expressed in various carcinomas (60–89% in breast, colon, and gastric) and hematological malignancies, yet masked by sialylation in healthy adult cells (18). Indeed, CD176 remains masked on healthy tissues due to terminal sialylation or carbohydrate chain prolongation, rendering it inaccessible and minimizing the risk of life-threatening “on-target/off-tumor” toxicity. The authors engineered two second-generation CD176-CAR constructs based on the specific monoclonal antibody Nemod-TF2. Experimental results demonstrated that these CD176-CAR-T cells successfully recognized CD176-positive cancer cell lines from various blood and solid tumor models, triggering robust T-cell activation, signaling, cytokine release, and target-specific cytotoxicity. The study concludes that targeting CD176 antigen offers a highly promising, specific, and safe immunotherapeutic strategy for treating a broad spectrum of hematological and solid malignancies while minimizing adverse effects on healthy tissues. In the context of highly refractory hematological malignancies, Cui et al. presented a pioneering case report on the use of CD38-specific CAR-T cells for chronic myeloid leukemia in blastic phase (CML-BP), where TKI resistance is common (19). Because CD38 is highly expressed on most acute myeloid leukemia (AML) blasts but is absent on healthy hematopoietic stem cells, it serves as an excellent target to eradicate resistant leukemic populations without permanently destroying the healthy bone marrow compartment. In the clinical study (NCT04351022), two CML-BP patients harboring multi-TKI-resistant mutations (E255K and T315I) achieved complete remission (MRD-negative) after receiving CD38-CAR-T cells following lymphodepletion. Notably, this therapy eliminated CD38^+^/BCR: ABL1^+^ blasts, suggesting that it successfully targets leukemic stem cells (LSCs) typically resistant to TKIs, while sparing the most primitive CD38^-^ hematopoietic stem cells (19). Ultimately, the findings highlight CD38-directed CAR-T cell therapy as a highly promising, novel immunotherapeutic strategy for overcoming multi-drug resistance and providing a bridge to allogeneic stem cell transplantation in patients with refractory CML-BP.

## Delivery and administration strategies for CAR-T cell therapy

One of the critical aspects associated with the efficacy of CAR-T cell therapy is the ability of the lymphocytes to migrate within the circulation to reach the tumor cells and their persistence. These features can also be affected by the methods used for the cell therapy administration to patients. Eylon et al., addresses the lack of a standardized venous access route for the administration of chimeric antigen receptor (CAR) T cell therapy in pediatric and young adult patients. Historically, there has been hesitation to use implanted mediports for cellular therapies due to theoretical risks of needle dislodgement and subsequent cell infiltration into the subcutaneous space. To investigate this, a retrospective study collected the results of the usage of mediport for the infusions (N = 504) of CAR-T cells into pediatric cohorts of patients from 34 clinical centers (N = 489 patients) and treated with CAR-T cells (*Pediatric Real-World CAR Consortium*) (20). Mediports are implanted through venous access ports in the upper chest and are commonly used to administer drugs, fluids and other medications to the patients. This devise allows easier access for the infusion, less risk of infection and improved quality of life for patients as compared to the classical tunneled venous catheters. This study evaluates the usage of Mediport for the infusion of commercially produced CAR-T cells in pediatric patients across the USA and represents the largest study with this aim. The goal of proving the feasibility and safety in utilizing this route of cell therapy infusions for patients who already have Mediports in place has been achieved (20).

## Understanding the predictors and clinical management of CAR-T cell therapy toxicities

CAR-T therapy for B-cell malignancies is highly effective but frequently complicated by severe toxicities such as cytokine release syndrome (CRS), neurotoxicity (ICANS), and B-cell aplasia. Clinical management relies on monitoring of early symptoms and the use of tocilizumab (anti-IL-6) or glucocorticoids to control high-grade inflammatory responses. To further optimize patient safety without compromising treatment efficacy, recent studies in this series delve into the molecular mechanisms that trigger these toxicities and evaluate the long-term impact of their standard clinical management. Loeffler-Wirth et al. (21) present a results of a single-cell RNA sequencing of the infusion products showing that the severity of ICANS correlates with specific transcriptomic signatures related to cell cycling, T-cell exhaustion, and the metabolic status of the CARs, rather than broad phenotypic differences (21). These findings suggest that the intrinsic cellular characteristics and genetic markers of the administered CAR-T product play a crucial role in triggering severe neurological side effects, providing valuable insights for predicting toxicity and refining the molecular design of future cellular therapies.

Fortunately, the clinical management of these severe inflammatory adverse events can be effectively handled with pharmacological interventions without sacrificing the treatment’s efficacy. A comparative study of patients with relapsed or refractory multiple myeloma either undergoing or not administration of glucocorticoids confirmed that the anti-inflammatory treatment does not compromise therapeutic outcomes (22). Patients receiving steroids achieved clinical response rates and complete response rates statistically equivalent to those who did not (response rate (ORR) and complete response rate (CRR) of 85% and 57.5%), proving that even cumulative doses used to treat toxicity do not interfere with the anti-tumor activity of the infused cells (22). These findings suggest that the safety profile of CAR-T products can be significantly improved by modulating T-cell function and refining cellular composition based on these genetic markers.

## Conclusions

Current cellular immunotherapy is transitioning from autologous models to versatile, “off-the-shelf” platforms to bypass logistical and biological barriers. Innovations demonstrate that gene-edited products can maintain potent anti-tumor activity without the risks associated with HLA mismatch. Moreover myeloid-based cellular therapies are under development, although still at an early stage. To address the challenges of solid tumors, such as the immunosuppressive TME and antigen heterogeneity, strategies including dual-targeting, identification of novel TAAs, moderate-affinity epitopes, and co-engineering with chemokine or immunomodulatory agents are being refined to improve the immune infiltration at tumor sites and reduce “off-target” toxicities.

Finally, clinical management is becoming more standardized through, on the one hand, the validated administration routes like Mediports and, on the other hand, the early onset identification of CRS and ICANS that are managed with glucocorticoids without compromising CAR-T efficacy. These advancements suggest that the path toward the development of more precise, effective and safer living drugs for both hematological and solid malignancies has been drawn, even through the integration with conventional treatments.

Adoptive Cell Therapy, Chimeric Antigen Receptor, Allogeneic Cell Therapies, Hematological Malignancies, Solid Tumors, Tumor-Associated Antigens, Gene Editing, Toxicities.
